# Nanoplastics Increase Fish Susceptibility to *Nodavirus* Infection and Reduce Antiviral Immune Responses

**DOI:** 10.3390/ijms23031483

**Published:** 2022-01-27

**Authors:** Carmen González-Fernández, Alberto Cuesta

**Affiliations:** Immunobiology for Aquaculture Group, Department of Cell Biology and Histology, Faculty of Biology, Regional Campus of International Excellence “Campus Mare Nostrum”, University of Murcia, 30100 Murcia, Spain; carmen.gonzalez1@um.es

**Keywords:** nanoplastics, fish, European sea bass, antiviral, *nodavirus* (NNV)

## Abstract

Nanoplastics (NPs) might cause different negative effects on aquatic organisms at different biological levels, ranging from single cells to whole organisms, including cytotoxicity, reproduction, behavior or oxidative stress. However, the impact of NPs on disease resistance is almost unknown. The objective of this study was to assess whether exposure to 50 nm functionalized polystyrene NPs impacts fish susceptibility to viral diseases both in vitro and *in vivo*. In particular, we focused on the nervous necrosis virus (NNV), which affects many fish species, producing viral encephalopathy and retinopathy (VER), and causes great economic losses in marine aquaculture. In vitro and in vivo approaches were used. A brain cell line (SaB-1) was exposed to 1 μg mL^−1^ of functionalized polystyrene NPs (PS-NH_2_, PS-COOH) and then infected with NNV. Viral titers were increased in NP-exposed cells whilst the transcription of inflammatory and antiviral markers was lowered when compared to those cells only infected with NNV. In addition, European sea bass (*Dicentrarchus labrax*) juveniles were intraperitoneally injected with the same NPs and then challenged with NNV. Our results indicated that NPs increased the viral replication and clinical signs under which the fish died although the cumulate mortality was unaltered. Again, exposure to NPs produced a lowered inflammatory and antiviral response. Our results highlight that the presence of NPs might impact the infection process of NNV and fish resistance to the disease, posing an additional risk to marine organisms.

## 1. Introduction

Plastic debris in the environment continues increasing as consequence of plastic production (estimated around 359 million tons of plastics per year; [1]), being an emerging concern. In the last decades, researchers have performed great efforts to identify the potential harmful effects of plastic debris by focusing on one particle type, microplastics (MPs; 5 mm to 100 nm in size), which are able to enter the trophic chain easily [2]. Nevertheless, with the improvement in generation and detection technologies, smaller particles have been revealed, i.e., nanoplastics (NPs; <100 nm in size) [3,4,5], which are highly reactive in the natural environment [6]. Plastic size has been revealed as one of the key parameters affecting plastic toxicity [7]. Unlike MPs, particles with lower sizes such as NPs have the capability to cross cellular membranes, resulting in high toxicity [8,9]. In addition to their own toxicity, the presence of MP/NPs in natural environments promotes their interaction with other factors. In seawater, it has been demonstrated that plastic particles are able to sorb into the polymer surface of several molecules, including hazardous hydrophobic contaminants [10], organic matter, and nutrients, forming an eco-corona [11,12,13]. In addition, plastic particles are able to sorb or be colonized by microorganisms, including pathogens, forming a biofilm, which has been called a “*plastisphere*” [14]. This *plastisphere* can include not only bacteria but also viruses, which makes them a potential vector for diseases [15]. Thus, plastic debris presents two main risks: (i) acts as a vector of pollution and pathogens and (ii) can be toxic to marine organisms, making them more susceptible to pathogen’s infections. The first hypothesis was recently reviewed [16], evidencing the attachment of several pathogens to plastic surfaces, among which *Vibrio* spp. were prevalent [17]. In fact, *vibriosis,* produced by several species of the genus *Vibrio*, towards viral encephalopathy and retinopathy (VER), caused by the nervous necrosis virus (NNV), is one of the most important bacterial and viral diseases in Mediterranean aquaculture [18]. However, few studies have attempted to elucidate if the exposure to NPs could impact the process of infection or animal susceptibility to different pathogens. Our first observations demonstrated that gilthead seabream (*Sparus aurata*) and European sea bass (*Dicentrarchus labrax*) exposed to MPs presented decreased immunity both in vitro and in vivo [19,20], probably caused by the increase of oxidative stress, which pointed to increased susceptibility to diseases. Exposed to polystyrene NPs, zebrafish (*Danio rerio*) larvae did not have altered susceptibly to *Aeromonas hydrophila* infection [21] while in orange-spotted grouper (*Epinephelus coioides*), reduced expression of toll-like receptor (TLR) and some interferon (IFN)-related genes occurred before and after NNV invasion, although fish mortality was not significantly affected [15]. Further studies are still needed to ascertain if the observed impaired immunity to mount an effective response might be linked to extended susceptibility to disease, as suggested.

NNV is a small non-enveloped virus with a diameter of around 25–30 nm and a genome composed of two single-stranded positive-sense RNA molecules, known as RNA1 (coding for the RNA-dependent RNA polymerase) and RNA2 (coding for the capsid protein) [22]. The genus *Betanodavirus* comprises four genotypes, with the RGNNV genotype the most widely distributed and that has the widest range of susceptible fish hosts [23]. Among them, European sea bass, a bony fish species of great economic importance for Mediterranean aquaculture, has shown sensitivity to numerous pathogens, being particularly susceptible to NNV, which causes up to 100% mortality in juvenile and larval stages [24]. NNV affects the central nervous system (brain, spinal cord, and eyes), impacting fish behaviour and swimming, leading to death. Taking into consideration actual knowledge, we evaluated in this study if the pre-exposure to functionalized polystyrene NPs can modify the immune response and susceptibility to NNV in fish using both in vitro and in vivo approaches. For that purpose, we used a battery of gene markers including chemokines (*cxcl9* and *cxcr3*), a macrophage marker (*csf1r*), inflammatory markers (*mpo*, *il8* and *il1b*), type I IFN (*mda5, irf3, pkr, mx* and *isg15*), and cellular stress (*nrf2* and *hsp70*) genes that have been demonstrated to be affected by NNV infection [25,26,27].

## 2. Results

### 2.1. Functionalized NPs Increase NNV Susceptibility of SaB-1 Cells and Alter Their Immunity

**Figure 1:**
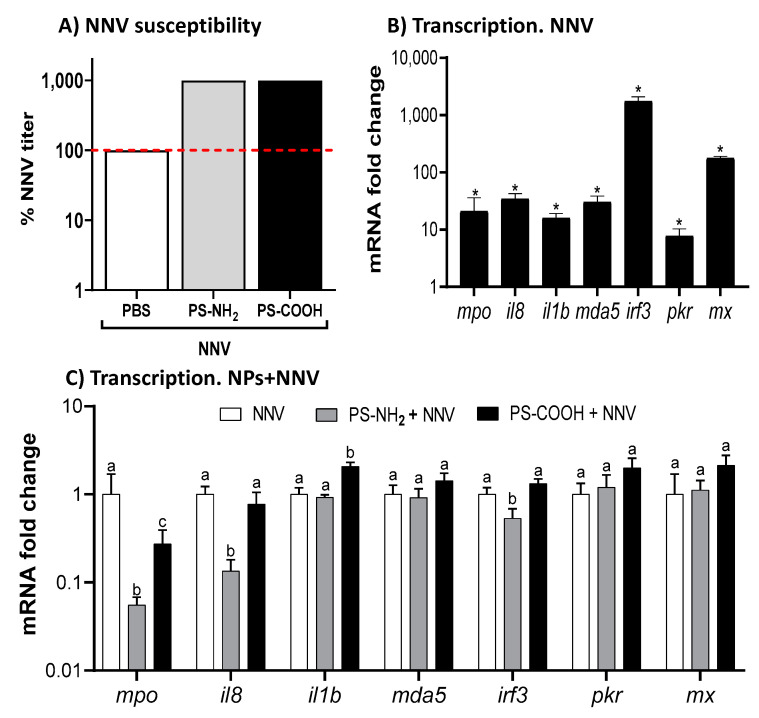
**Exposure to functionalized polystyrene nanoplastics (NPs) promotes viral susceptibility but slightly alters the transcriptional profile.** (**A**) Percentage of *nodavirus* (NNV) titer in SaB-1 cells exposed to 1 µg mL^−1^ of 50 nm functionalized polystyrene NPs (PS-NH_2_ and PS-COOH) for 24 h and then infected with NNV. The viral titer was determined after 10 days of infection, and the percentage of viral titer with respect to NP-unexposed (PBS) and NNV-infected cells was calculated. Red line indicates 100% of mortality with NNV treatment. (**B**) Transcriptional profile of SaB-1 cells infected with NNV for 24 h. Transcription is presented as the mean mRNA fold-change of the relative gene expression compared to mock-infected cells ± SEM (*n* = 3). Asterisks indicate significant differences between NNV- and mock-infected SaB-1 cells (Student-*t* test; *p* < 0.05). Values higher than 1 indicate up-regulation due to NNV infection. (**C**) Transcriptional profile of SaB-1cells pre-exposed to 1 µg mL^−1^ of PS-NH_2_ and PS-COOH for 24 h and then infected with NNV for 24 h. Transcription is presented as the mean mRNA fold-change of the relative gene expression compared to NNV-infected cells ± SEM (*n* = 3). Lowercase letters denote significant differences between NP-exposed and NNV-infected compared to NNV-infected cells (ANOVA and Tukey’s post-hoc tests; *p* < 0.05). Values higher than 1 indicate up-regulation due to NP exposure whilst those lower than 1 indicate down-regulation. ANOVA, analysis of the variance; *mpo*, myeloperoxidase; *il8*, interleukin 8; *il1b*, interleukin 1β; *mda5*, melanoma differentiation-associated gene 5; *irf3*, interferon regulatory factor 3; *pkr*, dsRNA-dependent protein kinase receptor; and *mx*, myxovirus (influenza) resistance protein.

Since the main target tissue for NNV infection is the brain, we used a fish cell line (SaB-1) derived from the brain of gilthead seabream to investigate the effects of NPs during infection at the cellular level. First of all, we aimed to investigate whether NPs promote NNV infection. For this, SaB-1 cells were exposed to NPs for 24 h and then infected with NNV, determining the cell cytopathic effect daily and the viral titer after 10 days of infection. Thus, pre-exposure to NPs increased cellular susceptibility to NNV since the final viral titer in cells exposed to NPs increased 10-fold compared to unexposed cells (Figure 1A). Once we confirmed the increased cellular susceptibility to NNV infection, we tested whether the SaB-1 immune response to NNV was modulated by NPs by evaluating the transcription of selected genes. To do this, we first showed that NNV infection significantly up-regulated the transcription of inflammatory (*mpo, il8* and *il1b*) and type I IFN (*mda5, irf3, pkr* and *mx*) genes (Figure 1B) compared with mock-infected cells, demonstrating that SaB-1 cells trigger the immune response and respond to NNV infection. Then, we tested if this immune response was modulated by pre-exposure to functionalized NPs. We found that pre-exposure to PS-NH_2_ significantly reduced *mpo, il8,* and *irf3* transcription upon infection compared with NP-unexposed but NNV-infected cells, while exposure to PS-COOH decreased and increased that of *mpo* and *il1b*, respectively (Figure 1C).

### 2.2. NPs Slightly Impacted the Immune System of European Sea Bass Juveniles

**Figure 2:**
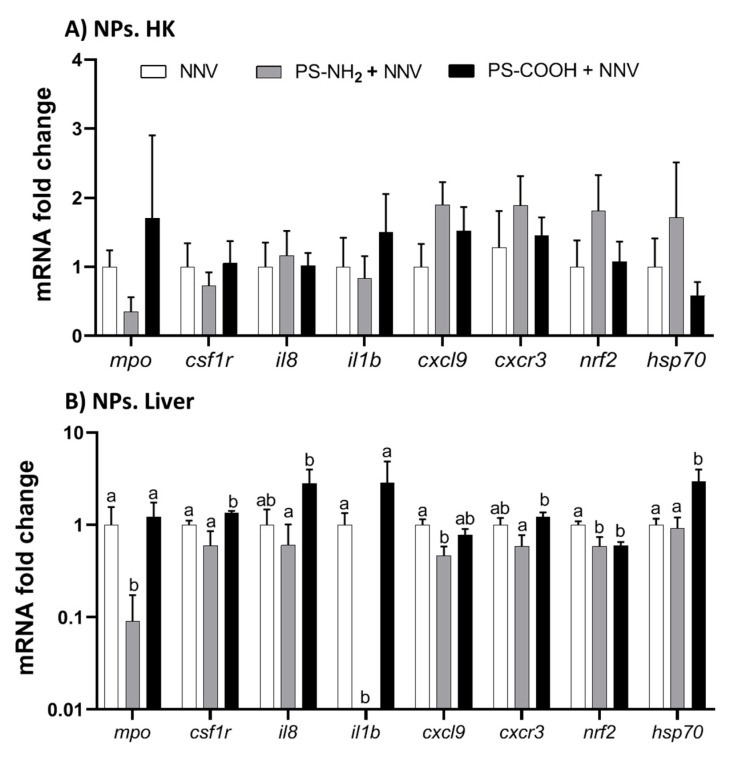
**Exposure to functionalized polystyrene nanoplastics (NPs) slightly alters the transcriptional profile in European sea bass.** European sea bass juveniles were intraperitoneally injected with phosphate buffer (PBS; Control) or with 1 μg mL^−1^ of 50 nm functionalized polystyrene NPs (PS-NH_2_ and PS-COOH), and the transcription profile in the head-kidney ((**A**); HK) and liver (**B**) was evaluated after 96 h by real-time PCR. Lowercase letters denote significant differences between NP-exposed and control treatments according to ANOVA and Tukey’s post-hoc tests (*p* < 0.05). *nrf2*, nuclear factor (erythroid-derived 2)-like 2; *csf1r*, macrophage colony-stimulating factor 1 receptor; *cxcl9*, CXC chemokine 9; *cxcr3*, CXC motif chemokine receptor 3; *hsp70,* heat-shock protein 70; and *isg15,* interferon-stimulated gene 15.

The intraperitoneal injection of functionalized NPs failed to alter the transcription of the selected genes in the head-kidney (HK; Figure 2A). By contrast, a significant down-regulation was observed in the liver for the *mpo, il1b, cxcl9,* and *nrf2* genes upon sea bass exposure to PS-NH_2_ whilst those exposed to PS-COOH showed a decreased transcription of *nrf2* and an increase in *csf1r* and *hsp70* genes (Figure 2B). Significant differences were also observed between NPs with respect to *mpo, csf1r, il8, il1b, cxcr3,* and *hsp70* genes.

### 2.3. Exposure to NPs Increases the Viral Load and Clinical Signs but Not the Mortality of European Sea Bass Juveniles

**Figure 3:**
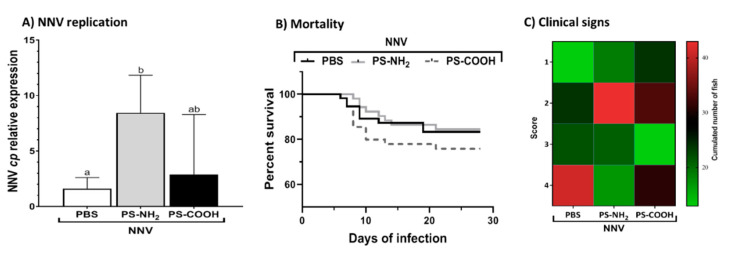
**Exposure to functionalized NPs reduced the clinical signs under which fish died upon infection with nervous necrosis virus (NNV).** European sea bass juveniles were intraperitoneally injected with phosphate buffer (PBS; Control) or with 1 μg mL^−1^ of 50 nm functionalized polystyrene NPs (PS-NH_2_ and PS-COOH) and 96 h later were challenged with NNV by intramuscular injection. (**A**) NNV replication in the brain of NNV-infected specimens was determined by real-time PCR three days post-infection. Lowercase letters denote significant differences between groups according to ANOVA and Tukey’s post-hoc tests (*p* < 0.05). (**B**) Kaplan–Meier survival curves showing the proportion of European sea bass survivors upon NNV challenge. (**C**) Heatmap presenting the cumulative number of fish showing disease signs based on their severity score: (1) changes in the colour of the skin, slower rhythm of swimming, and/or slower reaction to external stimuli such as feeding; (2) alterations in the swimming balance and/or erratic swimming spasms; (3) continuous erratic swimming; and (4) complete incapacity to maintain balance, swim, and/or move without external stimuli.

Once we confirmed the changes provoked by functionalized NPs in sea bass juveniles, we evaluated their impact on the susceptibility to NNV infection. First, we confirmed the NNV genome replication in the brain from NNV-infected sea bass juveniles (Figure 3A) and found that the viral replication of NNV was significantly increased in the brain of PS-NH_2_-exposed fish compared to those that were unexposed (Figure 3A). Regarding the susceptibility, the survival in juveniles unexposed to NPs was 83.3% but in those previously exposed to PS-NH_2_ or PS-COOH was 84.4 or 75.8%, respectively (Figure 3B), and no significant differences were observed. Fish challenged with NNV presented the typical signs of infection with a severity score of 4 in most cases before death (Figure 3C), whilst fish exposed to PS-NH_2_ or PS-COOH died upon NNV infection with main clinical scores of 2 or 2 and 4, respectively, indicating fish death with lower signs.

### 2.4. Exposure to NPs Alters the Immune Response against NNV

**Figure 4:**
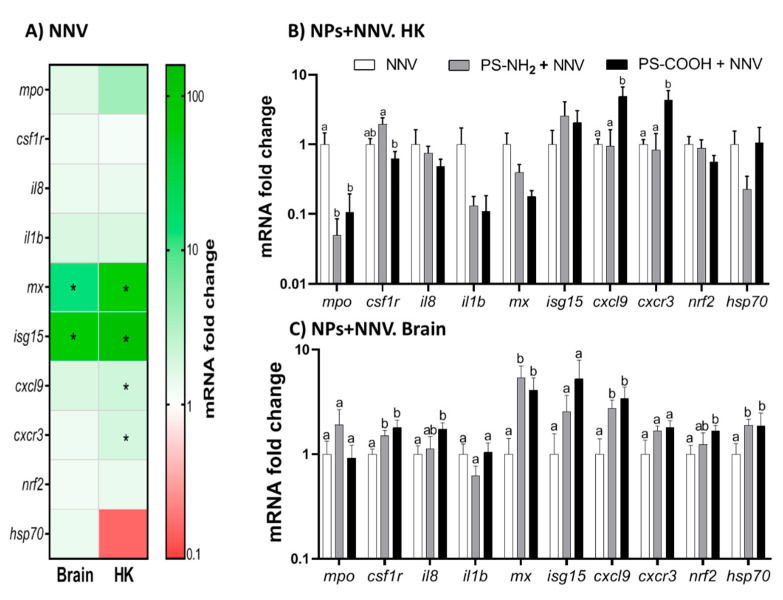
**Exposure to functionalized nanoplastics (NPs) differently alters the transcription induced by nervous necrosis virus (NNV).** European sea bass juveniles were intraperitoneally injected with phosphate buffer (PBS; Control) or with 1 μg mL^−1^ of 50 nm functionalized polystyrene NPs (PS-NH_2_ and PS-COOH) and 96 h later challenged with NNV by intramuscular injection and analysed by real-time PCR three days h post-infection. (**A**) Heatmap of the transcriptional profile in the brain and head-kidney (HK) of sea bass challenged with NNV. Data are presented as the mean mRNA fold-change of the relative gene expression in NNV-infected specimens compared to mock-infected ± SEM (*n* = 6). Asterisks denote significant differences between groups (Student-*t* test; *p* < 0.05). Transcriptional profile in the head-kidney (**B**) and brain (**C**) in NP-exposed and NNV-challenged fish. Data are presented as the mean mRNA fold-change of the relative gene expression in NP-exposed and NNV-challenged specimens compared to those only challenged ± SEM (*n* = 6). Lowercase letters indicate significant differences between treatments according to ANOVA and Tukey’s post-hoc tests (*p* < 0.05).

The transcription of several genes involved in defence, inflammation, and stress pathways was then analysed. Upon infection, we observed marked up-regulated transcription of the type-I IFN genes *mx* and is*g15* (Figure 4A), as the main antiviral genes, in the brain and head-kidney as well as the chemokine genes, *cxcl9* and *cxcr3*, in the head-kidney. Therefore, we evaluated whether this response to viral infection was affected by the exposure to functionalized NPs. In the head-kidney, down-regulation of *mpo, il1b,* and *mx* genes was registered in all NP-exposed fish upon infection, though only that of *mpo* reached significance (Figure 4B). By contrast, an up-regulation of the chemokines *cxcl9* and *cxcr3* was observed in those exposed to PS-COOH NPs (Figure 4B). In the brain, the target tissue for NNV, an up-regulation of genes coding for *csf1r, mx, cxcl9,* and *hsp70* was registered upon infection in all NP-exposed fish while the transcription of *il8* and *nrf2* was only induced in PS-COOH-exposed and NNV-infected specimens (Figure 4C).

## 3. Discussion

The aim of this study was to evaluate whether the pre-exposure to functionalized polystyrene NPs can modify the immune response and susceptibility to NNV in fish using both in vitro and in vivo approaches. Our data demonstrate that NPs increased SaB-1 cell susceptibility to NNV infection although the immune response was slightly altered. In vivo, European sea bass injection of NPs led to increased viral replication and clinical signs upon NNV infection, though mortality was not affected, as well as up-regulated gene transcription in the brain.

Viral infections, and, in particular NNV, are responsible for numerous fish mortalities and economic losses in aquaculture, being the major viral disease affecting Mediterranean aquaculture [18]. In this study, we used a brain cell line (SaB-1), which has been demonstrated to show good replication of the NNV virus [28], to study the mechanisms of infection and the immune response in a tissue-specific manner. In addition, we already showed that SaB-1 cells are susceptible to NP internalization [29], being a suitable model to study NP–cellular interactions. Cell lines are frequently used as experimental models due to their easy replicability and maintenance. In particular, fish cells lines have become useful tools for the ecotoxicity assessment and ranking of engineered nanomaterials [30]. Our results showed that cells exposed for 24 h to 1 µg mL^−1^ of functionalized plastics showed a 10-fold increase in virus replication, which is in agreement with a recent publication where GS cells infected with NNV displayed higher virus replication [15]. Surprisingly, and in sharp contrast to what was expected, the transcription profile of NNV-infected cells was slightly altered due to the previous exposure to NPs. In fact, cells exposed to PS-COOH showed certain up-regulation of inflammatory and IFN genes upon viral infection. Our results can be explained by the behaviour that the particles displayed in the cell culture medium. Commercial PS-NH_2_ and PS-COOH displayed positive and negative charges, respectively, in ultrapure water. However, once resuspended in the culture medium, all the particles reduced their ζ-potential and showed a similar negative charge very close to the medium alone (−9.1 ± 1.0 mV) [29]. This charge reduction could be associated with the adsorption of proteins and lipids in the culture medium [31]. The interaction of negative PS-NPs with cell membranes, also negatively charged, has recently been demonstrated thanks to the hydrophobic interaction and Van der Waals’ forces [32]. Particles of small sizes (around 50 nm) were internalized by RBL-2H3 cells through clathrin-mediated and caveolin-mediated pathways and micropinocytosis [32]. Clathrin-mediated endocytosis is also the primary route of internalization of virus into the cell [33]. Although we have not investigated the mechanism, we have reported that SaB-1 cells internalize NPs and suffer a strong and general stress state [29]. Therefore, the increased endocytic mechanism in cells leading to NP internalization might also contribute to increase the NNV attachment and entry, which has been suggested for GS cells [15]. If NPs and virus enter the cells, cellular mechanisms need to be activated to stimulate immune pathways to fight against stress and viral infection. The increased entry of NNV in NP-exposed cells may result in the increased final titers observed, in parallel with increased transcription of inflammatory and antiviral genes as a consequence of a higher number of viral genomes and particles. There are contradictory results on this issue. A zebrafish cell line exposed to PS-NPs (50 nm) and poly (I:C), a viral RNA agonist, showed increased transcription of antiviral genes in presence of NPs at low doses (5 µg mL^−1^) when compared to poly (I:C) alone [21]. By contrast, fish GS cells exposed to NPs (50 µg mL^−1^) and then challenged with viruses showed a significant reduction in the transcription of Toll-like receptors (*TLR1* and *TLR9*) and IFN (*IFNr* and *IFNh*) genes compared to those only infected [15]. This information may suggest that low concentrations of NPs are able to stimulate the expression of genes involved in antiviral responses while high concentrations seem to promote a down-regulation, but further observations are needed to reinforce this hypothesis.

Taking into account the scarce information available regarding the interplay between NP exposure and virus infection, we decided to perform an in vivo study to further investigate the impact of NPs in the process of NNV infection. In our experiment, we supplied NPs via intraperitoneal injection, which is expected to elicit greater and faster changes compared to exposure by trophic transfer since it promotes a direct interaction between NPs and the immune cells, as has been previously reported [34]. We failed to observe any changes in the gene expression related to the immune system upon NP exposure after 96 h in head-kidney, while some genes, mainly those related to inflammation, were sharply decreased in the liver. The poor negative effects may be directly related to the low doses used in this study in comparison with previous work: 0.1 μg g^−1^ (this study), 0.75 μg g^−1^ [15] or 111 μg g^−1^ [21] fish body weight. Nevertheless, after challenge with NNV, we observed a significant impact due to NP exposure. The behaviour responses against infection differed with respect to the NNV control group. Although we did not observe significant differences in the percentage of fish mortality, pre-exposure to PS-NH_2_ resulted in higher viral replication in the brain whilst in those exposed to PS-COOH, the disease signs before fish death presented lower severity levels (50% of the group showed a severity level of 2 before death), pointing to a greater susceptibility. A recent study demonstrated that viruses replicate faster and greater in the brain and spleen of orange-spotted grouper (*Epinephelus coioides*) juveniles exposed to NPs, thus accelerating fish mortality with respect to control treatments, but the final cumulative mortality was not altered [15]. The severity of disease seems to be related to the pathogen mode of infection since NP pre-exposure (5 and 50 µg mL^−1^) of zebrafish larvae did not impact the survival after infection with *Aeromonas hydrophila* [21]. Thus, data clearly suggest that NP exposure facilitates the early events of the disease but does not alter the final susceptibility.

Regarding immune responses against viral infection, NNV has been revealed to promote the expression of pro-inflammatory cytokines [35] in addition to the IFN pathway. In our study, overall, exposure to NPs reduced the up-regulation provoked by the NNV infection regarding the main IFN genes, namely, *mx* and *isg15*. These results are in agreement with those reported by Wang et al., [15], who documented the decrease in TLR and IFN genes in those exposed to NPs and challenged. Our data also show that NPs tended to reduce the proinflammatory response, indicated by *mpo* or *il1b*. This fact might suggest several immune processes such as mobilization of immune cells to other target organs, lower cell production or even a decrease in the activity of immune cells caused by the presence of NPs. We can speculate that, in our case, mobilization of cells to other target organs occurred considering the up-regulation of *cxcl9* and *cxcr3* in the head-kidney, which regulate immune cell migration, differentiation, and activation [26], towards increased transcription in the brain of the macrophage marker *csf1r* and the chemokines *il8*, *cxcl9,* and *cxcr3.* In addition, we also observed a certain increase in the oxidative stress markers in the brain (*nrf2* and *hsp70*) in NP-exposed fish that support the in vitro study. NPs are known to produce oxidative stress in several species [36], while it is known that NNV infection leads to increased heat-shock proteins, including Hsp70 [25,27], and oxidative stress [37]. Therefore, cellular stress is induced by both NPs and NNV and could regulate the whole immune response, but further studies are needed to clearly establish the precise crosstalk between the involved pathways. The increasing abundance of emerging contaminants in the ocean makes it necessary to understand the mechanisms of interaction with existing pathogens. Nowadays, there is limited information available on the interaction of NPs and infections caused by pathogens, particularly viruses. Further studies including those on other nanoplastics (chemical composition, size, shape, and modification properties), fish-pathogen models, and techniques (different -omics) are needed for clearer and wider knowledge of the fish–NP interactions at both cellular and organism levels as well as their environmental relevance for fish.

## 4. Materials and Methods

### 4.1. Fish Maintenance

Healthy juvenile specimens of European sea bass (10.2 ± 0.7 g body weight) were purchased from a hatchery (Alevines Guardamar, Alicante, Spain). The fish were maintained in a laboratory recirculating aquaculture system (RAS; 28% salinity, 22–26 °C and with a 12-h light: 12-h dark photoperiod) with suitable aeration and filtration and were fed daily with a commercial diet (Skretting, Burgos, Spain). The handling of the specimens was always performed in accordance with the Guidelines of the European Union Council (2010/63/UE) and the Bioethical Committees of the University of Murcia (reference REGA ES300305440012 and Permit Number A13210701).

### 4.2. Cell Culture

The SaB-1 cell line, derived from the brain of gilthead seabream (*Sparus aurata*) [28], was cultured in L-15 Leibowitz medium (ThermoFisher Scientific, Waltham, MA, USA) supplemented with 15% fetal bovine serum (FBS, ThermoFisher Scientific, Waltham, MA, USA) and antibiotics [29]. Cells were sub-cultured by routine trypsinization methods, and viability was always higher than 95%.

### 4.3. Nanoplastics

Two commercial polystyrene nanoplastics (PS-NP; 50 nm) functionalized with carboxyl (PS-COOH; Polysciences) or amine (PS-NH_2_; Bangs Laboratories, respectively) groups were used. The hydrodynamic size and aggregation of NPs were previously confirmed by dynamic light scattering (DLS) and electron microscopy in ultrapure water and complete culture medium, with sizes less than 100 nm and a PdI<0.2, indicating no aggregation in all cases [29].

### 4.4. Effects of Functionalized PS-NPs in the Immunity and Viral Susceptibility of the SaB-1 Cell Line

The impact of functionalized PS-NPs on gene expression and the susceptibility of the SaB-1 cell line upon NNV infection were determined. SaB-1 cells were seeded in 96-well microplates (Nunc) at 4 × 10^4^ cells well^−^^1^, reaching 80% confluence the next day. NPs were prepared in ultrapure water (1 mg mL^−1^), further diluted in culture medium, and finally added to the cells, which were exposed for 24 h to 1 μg mL^−1^ PS-NH_2_ or PS-COOH. Controls contained the same diluents without NPs. Then, the supernatant was discarded and cells incubated with 10-fold dilutions of NNV (strain It/411/96, genotype RGNNV) without serum for 2 h. Afterwards, unattached viruses were discarded, and cells were incubated with 100 μL of medium with 2% serum. Cultures were observed daily under a phase microscope, and cytopathic effects were monitored for 7–10 days. Finally, the tissue culture infective dose producing 50% lysis (TCID50 mL^−^^1^) was calculated for each sample. The results are expressed as % viral titer with respect to the mock-infected cells. Exposure and infection were done in triplicate, and the experiment was repeated twice.

For gene expression analysis, 7.5 × 10^4^ SaB-1 cells well^−^^1^ were seeded in 6-well microplates (Nunc, ThermoFisher Scientific, Waltham, MA, USA) and exposed for 24 h to PS-NPs as above. Cells were then infected at a 0.1 MOI of RGNNV for 24 h; the supernatant was aspirated and the total RNA isolated using a PureLink^®^ RNA Mini Kit (ThermoFisher Scientific, Waltham, MA, USA). All treatments were performed and analyzed in triplicate.

### 4.5. Effects of Functionalized PS-NP Exposure on Fish Immunity and Viral Susceptibility

One hundred and eighty juveniles of European sea bass were allocated in six individual and separated RASs, forming three groups, in duplicate (Figure S1). Fish (*n* = 60/treatment) were intraperitoneally injected with 1 mL of phosphate buffer (PBS; control) or 1 μg mL^−1^ PS-NH_2_ or PS-COOH. Fish (*n* = 6 fish/treatment) were euthanized after 96 h by an overdose of clove oil, and liver and head-kidney (HK) tissues from each fish were extracted and immediately frozen in TRIzol^®^ Reagent (ThermoFisher Scientific, Waltham, MA, USA) for later total RNA isolation following the manufacturer’s instructions. In order to evaluate the impact of functionalized PS-NPs on viral susceptibility, the remaining fish (*n* = 54 fish/treatment) were then infected by an intramuscular injection of 100 µL containing 10^6^ TCID_50_ NNV fish^−^^1^ (Figure S1). An additional group containing unexposed fish was injected with culture medium serving as an infection control. Fish (*n* = 6 fish/treatment) brain and HK were then sampled three days post-infection (dpi) for RNA isolation as above. Disease signs and mortalities were recorded daily in the remaining specimens for 30 dpi. Four ranks of disease signs were scored attending to their severity as follows: (1) changes in the color of the skin, slower rhythm of swimming, and/or slower reaction to external stimuli such as feeding; (2) alterations in the swimming balance and/or erratic swimming spasms; (3) continuous erratic swimming; and (4) complete incapacity to maintain balance, swim, and/or move without external stimuli. Percentage of survival was calculated and presented by the Kaplan–Meier method.

### 4.6. Gene Expression Analysis

Total RNA isolated from SaB-1 cells or sea bass tissues was treated with DNAse I (Promega, Spain) to remove genomic DNA, and the first-strand cDNA was synthesized by reverse transcription using SuperScript™ IV Reverse Transcriptase (ThermoFisher Scientific, Waltham, MA, USA). Real-time PCR was performed using a 7500 Fast Real Time PCR System (Applied Biosystems, MA, USA) and PowerUp™ SYBR™ Green Master Mix (Applied Biosystems, MA, USA). Reaction mixtures were incubated at 95 °C for 10 min, followed by 40 cycles of 15 s at 95 °C, 1 min at 60 °C, and finally 15 s at 95 °C, 1 min at 60 °C, and 15 s at 95 °C. Gene expression was corrected by the geometric mean of the housekeeping elongation factor 1 alpha (*ef1a*) and ribosomal protein S18 (*rps18*) gene expression. Relative mRNA quantities of the target in each sample were normalized to the expression of the reference genes and to the control group, with the 2^−∆∆Ct^ value presented (Pfaffl, 2001). Primers are listed in Table S1. Negative controls with no sample were always included in the reactions.

### 4.7. Statistical Analysis

Statistical analysis was performed using Graphpad Prism 8. Differences were considered significant at *p* < 0.05. Student’s t-test was carried out in the in vitro study to determine differences between NNV-infected and mock-infected cells. One-way-ANOVA was performed to establish significant differences in gene expression between NP-exposed specimens, as well as between NP-exposed plus NNV-infected cells or specimens. Normality and homogeneity of variances of the data distribution were tested, and Tukey’s post hoc test was used to test differences, if required, after one-way-ANOVA.

## 5. Conclusions

Our results showed that low concentrations of functionalized NPs increased virus replication and were able to reduce the immune response against NNV both in vitro and *in vivo*. However, although mortality was not affected by exposure to NPs, challenged sea bass juveniles died with less severe clinical signs, suggesting increased susceptibility, as indicated by the reduced IFN response. These findings are of great interest taking into account that natural episodes of infection with NNV occur in the Mediterranean Sea and provide new information about the impact of NPs on the health of marine fish.

## Data Availability

Not applicable.

## Supplementary Materials

The following are available online at www.mdpi.com/article/10.3390/ijms23031483/s1.
