# Supplementary material for: Nanoplastics Increase Fish Susceptibility to Nodavirus Infection and Reduce Antiviral Immune Responses
Source: Int J Mol Sci. 2022 Jan 27;23(3):1483. doi: 10.3390/ijms23031483 (PMC8836078; doi:10.3390/ijms23031483)
Supplement: Supplementary file 1 [file ijms-23-01483-s001.zip › ijms-1517614-sup.pdf]

**Table S1.** Primers used for analysis of gene expression by real-time PCR in this study.

| Protein Name | Gene | Target | Sequence (5'→3') | Acc. Number |
|---|---|---|---|---|
| NNV capsid protein | NNV cp | NNV | GACGCGCTTCAAGCAACTC<br>CGAACACTCCAGCGACACAGCA | D38636 |
| Ribosomal protein S18 | *rps18* | SaB-1 cells | CGAAAGCATTTGCCAAGAAT<br>AGTTGGCACCGTTTATGGTC | AM490061 |
| | | Sea bass | TTCCTTTGATCGCTCTTAACG<br>TCTGATAAATGCACGCATCC | AY831388 |
| Elongation factor 1α | *ef1a* | SaB-1 cells | CTTCAACGCTCAGGTCATCAT<br>GCACAGCGAAACGACCAAGGGGA | AF184170 |
| | | Sea bass | CGTTGGCTTCAACATCAAGA<br>GAAGTTGTCTGCTCCCTTGG | AJ866727 |
| Myeloperoxidase | *mpo* | SaB-1 cells | TTGGTCCAGACATCCTCG<br>ATGGGCAAAGCGGTAG | FM148574 |
| | | Sea bass | GAAGAGTGGGGCCTTTGTTT<br>CTGGGCCTCAGTGAAGACTC | DLAgn_00118340 |
| Interleukin 8 | *il8* | SaB-1 cells | GCCACTCTGAAGAGGACAGG<br>TTTGGTTGTCTTTGGTCGAA | AM765841 |
| | | Sea bass | GTCTGAGAAGCCTGGGAGTG<br>GCAATGGGAGTTAGCAGGAA | AM490063 |
| Interleukin 1β | *il1b* | SaB-1 cells | GGGCTGAACAACAGCACTCTC<br>TTAACACTCTCCACCCTCCA | AJ277166 |
| | | Sea bass | CAGGACTCCGGTTTGAACAT<br>GTCCATTCAAAAGGGGACAA | AJ269472 |
| Melanoma differentiation-associated gene 5 | *mda5* | SaB-1 cells | CATCGAGATCATCGAGGACA<br>CCAGATGTCGCTCTTGAAGG | HS988207 |
| | | Sea bass | AATTCGGCAATGGTGAAGTC<br>TCATTGGTCACAAGGCCATA | AM986362 |
| Interferon regulatory factor 3 | *irf3* | SaB-1 cells | TCAGAATGCCCCAAGAGATT<br>AGAGTCTCCGCCTTCAGATG | AM956899 |
| | | Sea bass | AGAGGTGAGTGGCAATGGTC<br>GAGCAGTTTGAAGCCTTTGG | CBN81356 |
| dsRNA-dependent protein kinase receptor | *pkr* | SaB-1 cells | TCCTTTGGAACCTCCCTACC<br>TCGAGGGGGAAATGTTGTAA | HS988732 |
| | | Sea bass | AGGGTCAGAGCATCAAGGAA<br>GACACCTTGCTGTCCCAGTC | FM008342 |
| Nuclear factor (erythroid-derived 2)-like 2 | *nrf2* | Sea bass | GTTCAGTCGGTGCTTTGACA<br>CTCTGATGTGCGTCTCTCCA | FP335773 |
| Macrophage colony-stimulating factor 1 receptor | *csf1r* | Sea bass | TTTCGGAAAGGTTGTTGAGG<br>TCTCATCTGAATGGGCACTG | KM225787 |
| CXC chemokine 9 | *cxcl9* | Sea bass | TCTGTCAGCTCGCCTTTCTG<br>TTCGTACTTGGACACGCACA | DLAgn_00012980 |
| CXC motif chemokine receptor 3 | *cxcr3* | Sea bass | ATCCTGTACGCCTTTGTGGG<br>GTCGGCAGACTCAGACCAAA | ENSDLAT00005001752.1 |
| Heat-shock protein 70 | *hsp70* | Sea bass | GATCGAGAGGATGGTGCAGG<br>TTGCCCTTCAGGTTCTCGTC | DLAgn_00212020 |
| Myxovirus (influenza) resistance protein | *mx* | Sea bass | GTATGAGGAGAAGGTGCGTCC<br>CTCTTCCCCGAGCTTTGGTC | AM228977, HQ237501, AY424961 |
| Interferon-stimulated gene 15 | *isg15* | Sea bass | ACCGTCCGCTGTCTATTGACTA<br>CAGATGCCCCAGCGAAACC | HG916840 |
