# Supplementary figures and images for: Nanoplastics Increase Fish Susceptibility to Nodavirus Infection and Reduce Antiviral Immune Responses
Source: Int J Mol Sci. 2022 Jan 27;23(3):1483. doi: 10.3390/ijms23031483 (PMC8836078; doi:10.3390/ijms23031483)

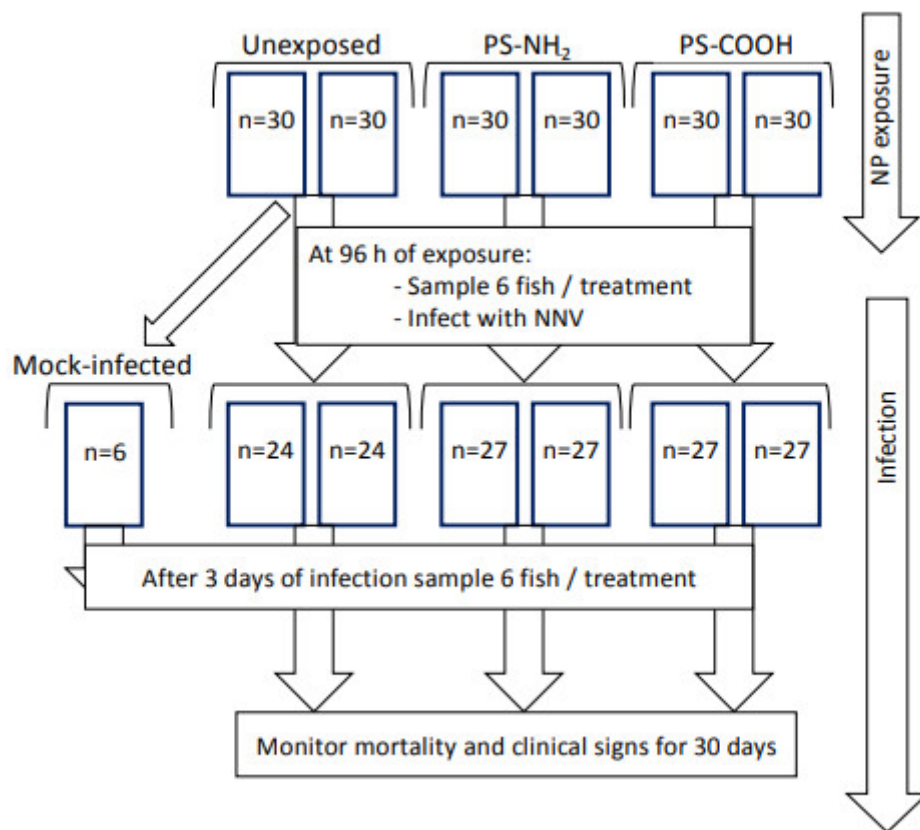

**Supplementary Figure S1.** Summary of the experimental design.

Supplement: Supplementary file 1 [file ijms-23-01483-s001.zip › ijms-1517614-sup figure S1.pdf]
